# Simultaneous intrathecal injection of muscimol and endomorphin‐1 alleviates neuropathic pain in rat model of spinal cord injury

**DOI:** 10.1002/brb3.1576

**Published:** 2020-03-18

**Authors:** Marjan Hosseini, Zohreh Karami, Mahmood Yousefifard, Atousa Janzadeh, Elham Zamani, Farinaz Nasirinezhad

**Affiliations:** ^1^ Department of Physiology School of Medicine Tehran University of Medical Sciences Tehran Iran; ^2^ Department of Physiology School of Medicine Iran University of Medical Sciences Tehran Iran; ^3^ Radiation Biology Research Center (RBRC) Iran University of Medical Sciences Tehran Iran

**Keywords:** central neuropathic pain, chronic pain, endomorphin‐1, muscimol, spinal cord injury

## Abstract

**Introduction:**

Due to side effects of medications used for chronic pain, combination therapy seems to be an appropriate solution for alleviation of chronic pain and reducing the side effects. The role of inhibitory GABA system is well proven in reducing neuropathic pain. Also, special attention has been focused on endogenous morphine (endomorphins) in reducing chronic pain originates from damage to the nervous system. The purpose of this study is to investigate the analgesic effect of simultaneous administration of GABA agonist and endomorphin‐1 on neuropathic pain in rat model of spinal cord injury (SCI). The role of oxidative stress, NR1 subunits of NMDA receptors, and α_2_ subunits of GABA receptors in the spinal cord has also been investigated.

**Methods:**

Spinal cord at level of T6–T8 was compressed. Three weeks after spinal cord injury, muscimol and endomorphin‐1 were injected (intrathecally once a day for 7 days) individually or in combination. Mechanical and cold allodynia, thermal and mechanical hyperalgesia were evaluated before injection and 15 and 60 min after injection. At the end of behavioral experiments, histological and biochemical evaluations were done on prepared spinal cord samples.

**Results:**

Isobologram results showed that combination therapy significantly increased the pain threshold comparing to injection of endomorphin‐1 (EM) or muscimol alone. Histological studies indicated the increased expression of α2 subunits of GABA receptors, and NR1 subunits of NMDA receptors in the spinal cord. The combination therapy also increased the glutathione (GSH) and superoxide dismutase (SOD) level and decreased the malondialdehyde (MDA) levels in the spinal cord.

**Conclusion:**

Simultaneous administration of muscimol and endomorphine‐1 could be a new candidate for alleviation of pain resulting from spinal cord injury.

## Graphical abstract

Simultaneous intrathecal administration of muscimol and endomorphin‐1 as a new candidate for the alleviation of pain resulting from SCI.

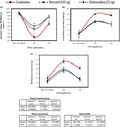

## 1 INTRODUCTION

Neuropathic pain which is caused by nerve irritation, damage, or destruction of the nervous system, is one of the six classes of chronic pain (Nasiri Nejad & Manaheji, 2003; NasiriNezhad & Sagen, 2005), and characterized by an abnormal sensation called dysesthesia, exaggerated pain sensation (hyperalgesia), and pain produced by normally nonpainful stimuli (allodynia) (Gajavelli, Castellanos, Furmanski, Schiller, & Sagen, 2008; Hama, Basler, & Sagen, 2006). Mechanism of neuropathic pain is not well understood but it seems that peripheral or central mechanism (e.g., increased activity of the excitatory pathways or decreased activity of inhibitory pathways) is involved.

GABA is a major inhibitory neurotransmitter that regulates the excitability of neurons in the mammalian central nervous system (CNS). Following SCI, the concentration and efficacy of this neurotransmitter reduce and lead to the address of an intensified pain signaling to higher areas (Gwak & Hulsebosch, 2011; Yowtak et al., 2013). GABA has three different receptors: GABA A and GABA C receptors are ionotropic and open the chloride ion channels; fast excitatory postsynaptic potentials are blocked by the agonist of these GABA receptors. GABA B receptor is metabotropic type and coupled to G‐proteins. This receptor reduces calcium inflow or potassium outflow across the cell via the second messenger or reducing the level of intracellular CAMP; slow excitatory postsynaptic potentials blocked by agonists of this GABA receptor (Watanabe et al., 2002). Muscimol is a GABA A receptor agonist and a local anesthetic extracted from “Amanita muscaria” mushroom. (Johnston, 2014).

The endomorphins are a group of endogenous opioid peptides consist of endomorphin‐1 and endomorphin‐2. They are produced in the brain, spinal cord, and possibly other tissues (Hollt, 1986; Zöllner & Stein, 2006). This opioids block pain transmission in the spinal cord through pre‐ and postsynaptic mechanisms (Chen & Pan, 2006). Moreover, they reduce pain by increasing the activity of inhibitory GABAergic interneurons and inhibit the perception of pain in the somatosensory cortex (Granmo, Jensen, & Schouenborg, 2013).

Central sensitization is another neuropathic pain mechanism, and several factors are involved in the creation of this phenomenon. Glutamate is a major excitatory neurotransmitter in the nervous system and contributes to 75% of CNS stimulations. NMDA receptors of Glutamate are inotropic and have high permeability to calcium and sodium ions. These receptors play a key role in creating and promoting of central sensitization (D'Angelo et al., 2013; Manresa et al., 2014). In this pathway, the activity of NMDA receptors has been greatly increased, resulting in more sodium and calcium influx followed by increasing excitability of neurons (Granmo et al., 2013; Willis, 1985). This pathway is inhibited by GABAergic neurons; therefore, reducing the activity of GABAergic neurons that occur following SCI can be a reason for central sensitization (Cao et al., 2011). Moreover, excessive stimulation of the NMDA receptor and entry of calcium ions causes excessive production of free radicals and stimulates the enzymatic processes that will lead to cell death (Xu et al., 2012).

Many studies indicate that free radicals are involved in the development of neurodegenerative diseases (Jiang, Akopian, Ho, Walsh, & Andersen, 2000; Tang & Aizenman, 1993; Yowtak et al., 2013). In addition, reactive oxygen species (ROS) are involved in reducing excitability and degeneration of GABA‐secreting neurons (Yowtak et al., 2013). This coincides with a reduction in the number of inhibitory neurotransmitters that are involved in hyperalgesia after neuronal damage. Any process that disturbs the balance between the production and destruction of free radicals can cause irreparable damage to the nervous system. Glutathione and superoxide dismutase are the most important endogenous antioxidants that play a key role in creating and maintaining the redox balance in the central nervous system. Cellular redox balance is essential for many internal processes including regulation of NMDA receptor activity. So, reducing agents increases the activity of this receptor, while oxidizing agents will decrease its activity (Bodhinathan, Kumar, & Foster, 2010; Tang & Aizenman, 1993).

Some medications are currently used to reduce chronic pain caused by SCI, but long‐term use of these drugs has many side effects, and the majority of sufferers do not usually response efficiently to routine therapeutic prescriptions (Mehta et al., 2012; Siegan, Hama, & Sagen, 1997). Sometimes combination of two or more drugs can lower dosage and negative side effects. Moreover, since patients with chronic pain have numerous symptoms, use of multiple drugs with different therapeutic function can improve different clinical manifestations (Mao & Gold, 2011). Combination therapy is a new and growing approach to the treatment of chronic pain and can be more effective than taking a medication individually. In the present study, we evaluated the therapeutic effects of endomorphin‐1 and muscimol on neuropathic pain threshold in a rat model of SCI.

## 2 MATERIALS AND METHODS

### 2.1 Animals

In this study, male Sprague–Dawley rats (140–160 g) were housed in plastic cages at a room with controlled temperature (23 ± 2°C) and humidity (50 ± 10%), maintained on a 12‐hr light–dark cycle with ad libitum access to food and water. All procedures were approved by the institutional animal ethics committee. Animals were randomly divided into 7 groups (*n* = 8): intact animals (control), sham‐operated (sham), animals with spinal cord injury (SCI), SCI animals treated with normal saline (SCI + vehicle), SCI animals treated with endomorphin‐1 (SCI + EM), SCI animals treated with muscimol (SCI + muscimol), and SCI animals treated with a combination of muscimol and endomorphin‐1 (SCI + combination).

### 2.2 Induction of neuropathic pain

Neuropathic pain was induced by compression of the spinal cord under general anesthesia with an intraperitoneal injection of ketamine (80 mg/kg) and xylazine (10 mg/kg). The skin in the thoracic vertebral column area was clean‐shaven and scrubbed with povidone–iodine. The spinal cord was exposed through an incision on the midline, and laminectomy at T6–T8 segments of the spinal cord was performed. The spinal cord is clamped by a micro clamp for 1 min. Then, wound was sutured and topical antibiotic was sprinkled. After full recovery from anesthesia, animals were kept in group cages for a 21‐day recovery. Because of urinary retention after SCI, bladder massage was done twice a day.

### 2.3 Drug treatment

Three weeks after SCI, a PE‐10 cannula was inserted into the subarachnoid space through L5‐L6 segments of the vertebral column under general anesthesia with a mixture of ketamine and xylazine. Muscimol and endomorphin‐1 (Sigma‐Aldrich), as well as normal saline, were injected with a Hamilton syringe in respective groups, three days after cannula fixation. The optimal dose of muscimol (0.01 µg/10 μl) and endomorphin‐1 (2/5 µg/10 μl) was administered for consecutive 7 days according to our pilot studies. Complete behavioral surveys were conducted to determination optimum dose of muscimol between 0.01, 0.1, and 1 µg also between doses of 2.5, 1, and 2 µg of endomorphine (Dose–response studies).

### 2.4 Behavioral studies

Mechanical and cold allodynia, as well as mechanical and thermal hyperalgesia, were evaluated before, 15 and 60 min after the last injection. Basso, Beat‐tie, and Bresnahan (BBB) scoring was used to assess the motor function of rats. Based on similar studies, we decided to choose between 15, 60, and 180 min to determine the most appropriate time for drug efficacy. In fact, in order to get the best effectiveness time, we selected the minimum effective time after the injection (15 min) based on studies and then determined how long it would be effective after the injection.

### 2.5 BBB

Postinjury locomotor capacity was assessed via the BBB locomotor scale method. The BBB score is used for function recovery and locomotor testing in chronic SCI study. The scale (0–21) represents sequential recovery stages and categorizes combinations of rat joint movement, hind limb movements, stepping, forelimb, and hind limb coordination, trunk position and stability, paw placement, and tail position. Each rat was placed in a clear box and stimulated to move. Their movement was controlled with 2 evaluators. Each evaluator made a determination of the locomotor capacity of the rats using the BBB functional scale.

### 2.6 Von Frey

To measure the mechanical allodynia, Von Frey monofilaments were used. The apparatus consists of an elevated horizontal wire mesh stand with a Plexiglas box set on top of the wire grid. The box was covered with perforated lids. Opaque separators were used to diminish interactions between rats. Animals were habituated for 15 min in the box; then, 6 calibrated nylon filaments (diameters of 4.08, 4.31, 4.56, 4.74, 4.93, 5.18 mm) were perpendicularly applied to the plantar surface of the rat hind paws. Each application was repeated 5 times for each filament to determine the mechanical threshold. The pressure increased gradually and linearly until a clear withdrawal of the paw was observed. The evaluation was stopped in the case of three positive responses, but if no response was elicited, the next higher filament was applied.

### 2.7 Acetone test

The rats were placed in a transparent plastic cage with a wire mesh floor for 20 min. Then, for assessment of cold allodynia, a drop of acetone squirted onto the midplantar surface of the hind paw. Brisk withdrawal, licking or biting of the hind paw was defined as a positive response. The test repeated 5 times with an interval of 5 min for each paw.

### 2.8 Randall–Selitto paw‐pressure test

To assess mechanical hyperalgesia, increasing pressure was gradually applied to the right hind paw using analgesia meter (Ugo Basile). At this test, with pressing a pedal switch the force is applied to the animal paw, which is placed on a small plinth under a cone‐shaped pusher with a rounded tip, which does not hurt the animal. When the rat struggled, the pedal was released and read off the force scale at which the animal felt pain. Additional weights were provided, to increase the force range. This method allows determining the threshold by vocalization response to mechanical nociceptive stimulation.

### 2.9 Histological studies

After behavioral tests, histological studies were made on some spinal cord samples for confirmation of spinal cord injury. The rats were deeply anesthetized with ketamine and xylazine. Transcardially perfusion of 0.2 M phosphate buffer saline (PBS) and aldehyde solution containing 4% paraformaldehyde and 0.2% glutaraldehyde in 0.2 M PBS (pH = 7.4) was made to fix the spinal cord tissues. Then, by a dorsal incision over the thoracic region and laminectomy, the spinal cord was exposed and the T6–T8 segments were removed according to the rat anatomical landmarks and kept in plastic containers filled with 4% paraformaldehyde at ambient temperature for 24 hr. For cryoprotection, the segments were immersed in 25% sucrose solution. By using cryostat microtome, 20‐µm tissue sections were prepared and air‐dried for 5 hr. They were dewaxed and stained with Cresyl violet for 1 min. Dehydration was finally performed with graded alcohol.

### 2.10 Biochemical studies

For biochemical studies, some rats in each group were randomly chosen and their spinal cords were dissected under deep anesthesia and rapidly frozen on liquid nitrogen and then stored at −80°C until assay.

### 2.11 Western blotting

Spinal cord tissues, 4 animals from each group, were homogenized in 200 μl ice‐cold lysis buffer (RIPA Lysis Buffer) with an electric homogenizer, rinsed the blade twice with another 200 μl lysis buffer, and then centrifuged at 13,000 × *g* at 4°C for 20 min. Supernatants were taken and stored at −80°C until Western blotting. To investigate protein expression, 2.5 μl of lysate was removed to perform a protein quantification assay, and using a plate reader, the protein concentration of 150 μg was determined for each cell lysate. Changes in protein expression of α2 subunit of GABA A receptor [(abcam, ab72445), Concentration used:1/1000], secondary antibody [(abcam, ab97200 Concentration used: 1/5000] and NR1 subunit of NMDA receptor [(abcam, ab52177), Concentration used:1/1000], and secondary antibody [(abcam, ab ab205718 Concentration used: 1/50000] were assessed with Western blotting, according to blotting protocol.

The selected housekeeping gene was β‐actin, and antibeta‐actin antibody (ab8227) was used to detect it. We used TotalLab V1.11 software to analyze the data. Relative intensities of the bands of interest were analyzed with TotalLab V1.11 software (Nonlinear Dynamics). The gene expression level (α2 subunit of GABA A and NR1 subunit of NMDA) was determined semiquantitatively by calculating the ratio from the target gene in relation to internal standard (β‐Actin).

### 2.12 Evaluation of oxidative stress

To evaluate levels of SOD, GSH, and MDA, tissue preparation, 4 animals from each group, and extraction of protein were performed as described earlier for Western blot.

### 2.13 Measurement of superoxide dismutase

Superoxide anion converts to hydrogen peroxide and oxygen by superoxide dismutase. The SOD activity was measured by the Worthington method. 0.1 M EDTA in 0.3 mM of sodium cyanide and 1.5 mM NBT added to sample in a Covet and vortexed for 5 min at 37°C. Then, 0.12 mM riboflavin in 0.067 M potassium phosphate buffer (pH = 7/8) was added and placed at room temperature for 10 min. Absorption at a wavelength of 560 nm was read within 5 min, and specific activity was calculated based on units of milligram protein.

### 2.14 Measurement of glutathione

Glutathione is an antioxidant that protects cell components from ROS, such as free radicals and peroxides. The Dietz method was used to determine tissue GSH level. The samples were mixed with 5% sulfosalicylic acid and centrifuged at 2000 *g* at 4°C for 10 min. A total of 100 µl of the supernatant was added to 810 µl of 0.3 M disodium phosphate. The reaction was started with adding 90 µl of 0.04% DTNB reagent in 0.1% sodium citrate. Absorbance amount at 412 nm was read within 5 min. Using 1 mg/µl GSH solution, standard curve plotted and the concentration of GSH in the samples was calculated.

### 2.15 Measurement of malondialdehyde

Malondialdehyde (MDA) level as a lipid peroxidation index is used to indicate the extent of damage caused by ROS. MDA reacts with thiobarbituric acid, and red color is absorbed in wavelength of 532 nm. To measure MDA, 250 µl of samples was added to 10 µl of butylated hydroxytoluene‐1; then, 250 µl of 1 M phosphoric acid and 250 µl of thiobarbituric acid were added to microtubes. The samples were vortexed and incubated at 60°C for 60 min. In the next step, the samples were centrifuged in 10, 000 *g* for 2–3 min and absorbance was read at a wavelength of 532 nm with a spectrophotometer. The standard curve was created based on various concentrations of tetramethoxypropane, and the optical density of the samples was adapted on the standard curve.

### 2.16 Statistical analysis

All results were shown as means ± *SEM*. The results were analyzed for statistical significance by one‐way ANOVA test and Tukey's post hoc multi‐comparison. A two‐way ANOVA test and Bonferroni post hoc were applied for the time course (comparison of before vs. after treatment). Repeated measures ANOVA was carried out to change the pain over time among before, 15, 60, and 180 min after treatment to clear the time of peak effect. In all calculations, *p*‐value < .05 was taken as significant.

## 3 RESULTS

### 3.1 Locomotor function after spinal cord injury

**Figure 1:**
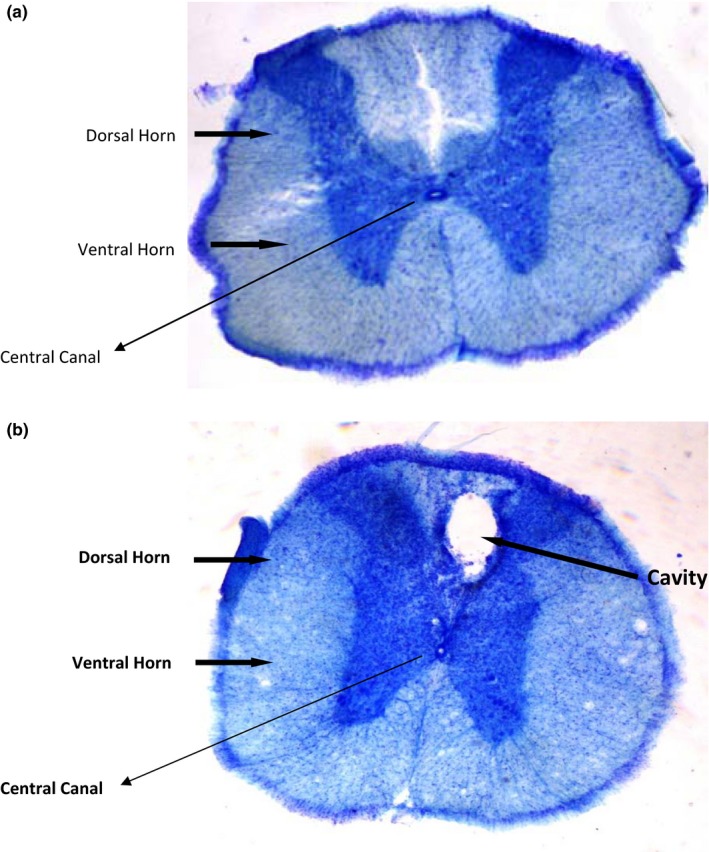
T6–T8 segments of the spinal cord. Compression of the spinal cord led to the creation of a cavity in the spinal cord. (a) control group. (b) SCI group

As shown in (Figure 1), compression of spinal cord led to the creation of a cavity in the spinal cord. Locomotor function was assessed after SCI for 4 min using the BBB locomotor rating scale.

**Figure 2:**
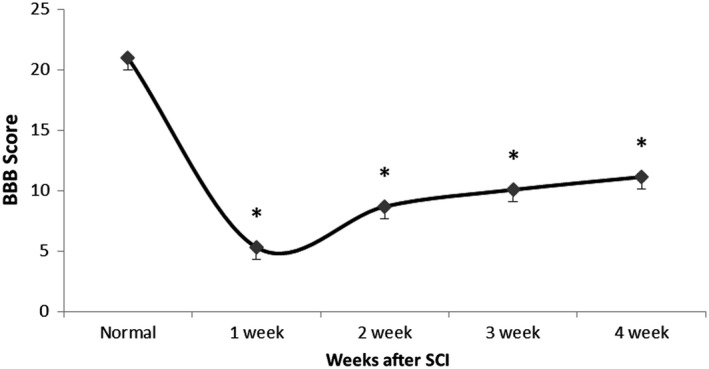
BBB scale, locomotor function significantly decreased after SCI. Normal: before SCI. *p < .001. First week was 5.313, Second week 8.688, third week 10.073, and fourth week 11.135

The average of this test for SCI group in the first week was 5.313, second week 8.688, third week 10.073, and fourth week 11.135. According to the BBB scale, locomotor function in SCI group significantly decreased *p* < .001 (Figure 2).

### 3.2 Pain threshold after combination therapy

**Figure 3:**
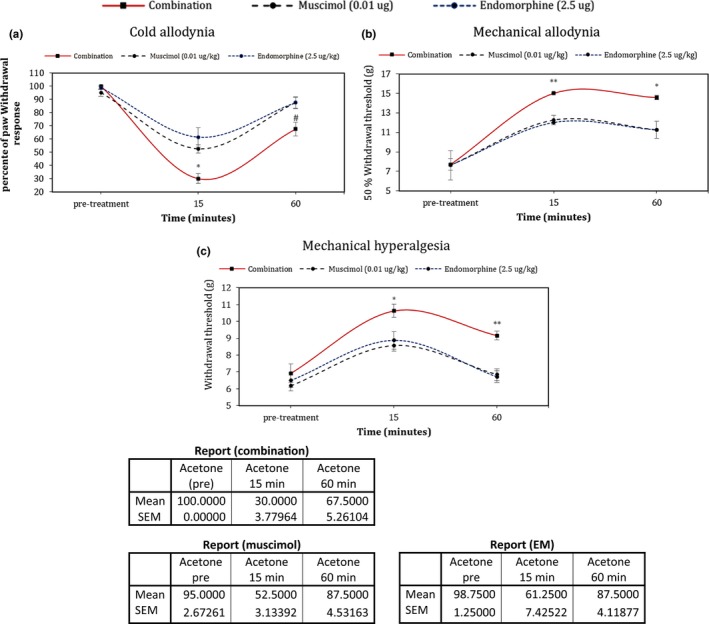
Effect of 7 days' intrathecal administration of muscimol, Endomorphin‐1, and the combination of these two drugs on symptoms of neuropathic pain. (a) Cold allodynia (Acetone test), (b) mechanical allodynia (Von Frey test), and (c) mechanical hyperalgesia (Randall–Selitto paw‐pressure test). Data presented as mean ± *SEM*. Pain threshold 15 and 60 min after Combination therapy significantly increased. Administration of vehicle (saline) was no different from pretreatment (data not shown). *p < .05, **p < .01, ***p < .001 significant difference between SCI + combination group with SCI + muscimol and SCI + EM groups. ^#^Significant difference at *p* < .001 level with same time compared to muscimol and endomorphine alone

The analgesic effect of 7 days' intrathecal administration of muscimol and endomorphin‐1 individually and in combination was assessed 15 and 60 min after the last injection in the 7th day, in respective groups. Cold (*df*: 21,2; *F* = 6.13; *p* < .05) and mechanical (in 15 min, *df*: 21,2; *F* = 18.5; *p* < .001), (in 60 min, *df*: 21,2; *F* = 7.2; *p* = .004) allodynia evaluation, showed that pain threshold at 15 min after the last injection in the SCI group that received a combination of drugs has significantly increased in comparison with other groups. In addition, this effect was remained 60 min after the last injection. Pain threshold changes in mechanical hyperalgesia (in 15 min, *df*: 21,2; *F* = 6.63; *p* = .006), (in 60 min, *df*: 21,2; *F* = 6.63; *p* < .05) assessment represented the same results (*p* < .001; Figure 3).

### 3.3 GABA A receptor's α2 subunit expression after combination therapy

**Figure 4:**
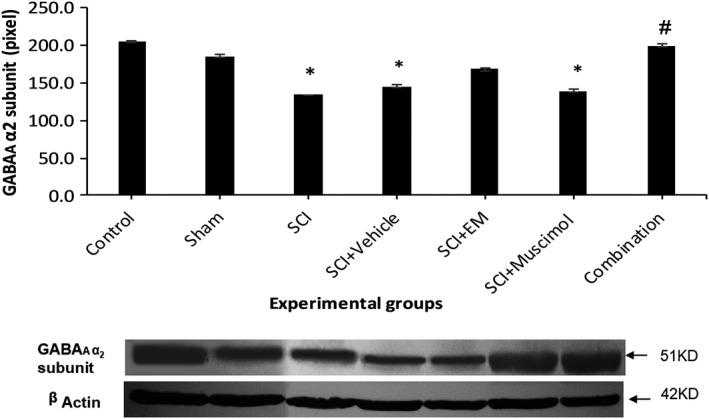
GABA A receptor's α2 subunit expression in Western blot. *Significant difference at *p* < .001 in comparison with control group. ^#^Significant difference at *p* < .001 in comparison with the SCI group. Control: 205.3, Sham: 185.1, SCI: 133.864, SCI + Vehicle: 145.146, SCI + EM: 168.059, SCI + Muscimol: 139.018, SCI + Combination: 199.308

As indicated in (Figure 4), SCI has significantly reduced the amount of GABA A receptor expression compared to control and Sham group (*p* < .001). Administration of muscimol, in SCI + muscimol group, did not have a significant effect on increasing α2 subunit expression, but as shown in (Figure 4), the level of α2 subunit expression significantly increased in SCI + EM and combination therapy groups compared to SCI group (*p* < .001).

### 3.4 NR1 subunit expression after combination therapy

**Figure 5:**
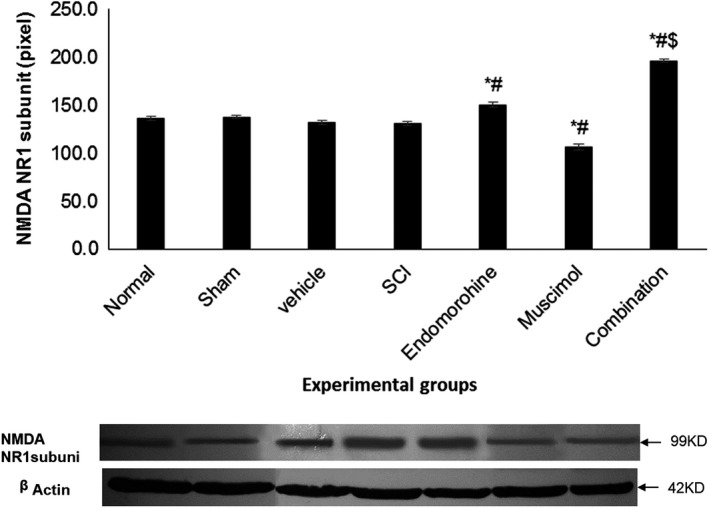
NMDA receptor's NR1 subunit expression in Western blot. *Significant difference with the control group at *p* < .05; ^#^Significant difference with SCI group at *p* < .05. ^$^Significant difference between the combination group and the other groups at the *p* < .001 level. Control: 136.45, Sham: 137.614, SCI: 131.021, SCI + Vehicle: 131.764, SCI + EM: 150.485, SCI + Muscimol: 106.636, SCI + Combination: 196.422

In this study, SCI did not have any effect on the expression of NR1 subunit of NMDA receptor in comparison with the control group, and on the other hand, it was found that the prescription of drugs did not reduce the level of expression of NR1 subunit in respective groups. This is while muscimol could reduce the level of expression of this subunit significantly compared the SCI group (*p* < .001; Figure 5).

### 3.5 Effect of combination therapy on oxidative stress

**Figure 6:**
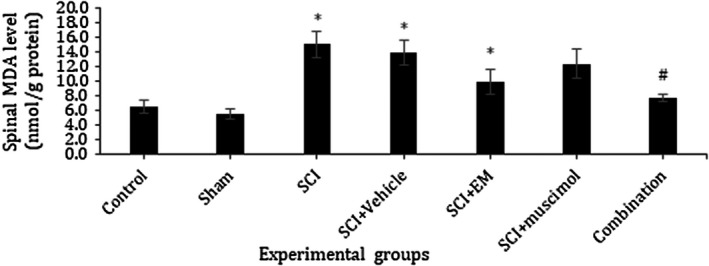
Effect of 7 days' intrathecal administration of muscimol, Endomorphin‐1, and the combination of these two drugs on level of MDA in the spinal cord tissue. *Significant difference at *p* < .05 with the control group; ^#^Significant difference at *p* < .05 with SCI. Data presented as mean ± *SEM*. Control: 6.466, Sham: 5.436, SCI: 15, SCI + Vehicle: 13.88, SCI + EM: 9.793, SCI + Muscimol: 12.290, SCI + Combination: 7.703

**Figure 7:**
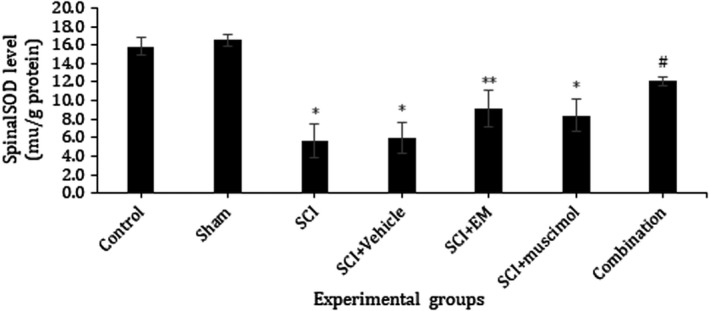
Effect of 7 days' intrathecal administration of muscimol, Endomorphin‐1, and the combination of these two drugs on level of SOD in the spinal cord tissue. *Significant difference at *p* < .05 with the control group, **Significant difference at *p* < .01 with the control group, and ^#^significant difference at *p* < .05 with SCI group. Data presented as mean ± *SEM*. Control: 15.866, Sham: 16.530, SCI: 5.663, SCI + Vehicle: 5.966, SCI + EM: 9.130, SCI + Muscimol: 8.380, SCI + Combination: 12.090

**Figure 8:**
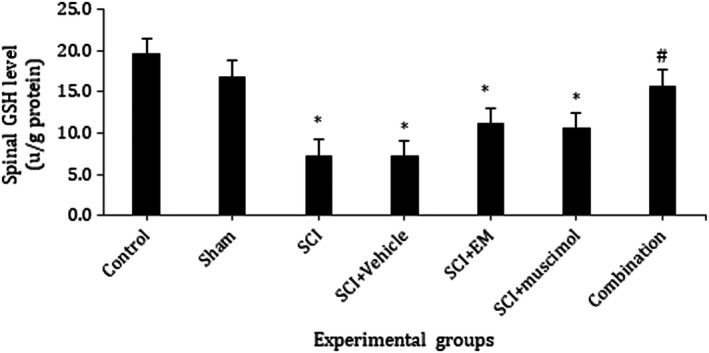
Changes in the level of GSH in the spinal cord tissue.*Significant difference at the *p* < .05 with the control group; ^#^significant difference at *p* < .05 with SCI. Data presented as mean ± *SEM*. Control: 19.66, Sham: 16.936, SCI: 7.34, SCI + Vehicle: 7.296, SCI + EM: 11.176, SCI + Muscimol: 10.613, SCI + Combination: 15.773

In this study, effects of combination therapy on the spinal cord, GSH and SOD activities, as well as MDA level, were assessed. Our results showed a significant increase in MDA level in the SCI group as compared with the control and sham group. This increase was attenuated by treatment with muscimol and endomorphin‐1 in combination (*p* < .05; Figure 6). When compared with the sham and control group, the activities of antioxidant enzymes GSH and SOD were significantly decreased in SCI animals. Treatment with muscimol and endomorphin‐1 in combination and individually markedly increased GSH and SOD activity when compared with the SCI group (*p* < .05; Figures 7 and 8).

## 4 DISCUSSION

Neuropathic pain occurs after primitive damage or impairment of neuronal function, in the peripheral or central nervous system. In recent years, in order to investigate the mechanisms of this type of pain and the effect of different therapeutic agents on it, several animal models have been proposed, in which the SCI model is more acceptable for its similarity to clinical manifestations of the disease in human. One of the methods used by the researchers in the last decade is the use of vascular micro clamping (Kerimoğlu et al., 2007). The advantage of this method is that the risk of infection after the lesion is low, resulting in a reduction in the mortality rate due to the possibility of better care and also the specificity and precision of the lesion site (Liang & Gross, 1999).

Today, drugs that are commonly used to relieve pain have unwanted side effects, especially in the pain‐tolerant dose and the combination of these drugs can reduce the administered dose (Attal et al., 2010; Mao & Gold, 2011). In the present study, the effect of combination therapy with intrathecal administration of muscimol and endomorphin‐1 as a GABA and μ‐receptor agonist was investigated in a rat model of SCI.

The symptoms and signaling pathways of neuropathic pain are multiple and complex. The most important symptoms are abnormal responses to stimuli, such as allodynia and hyperalgesia, which can be evaluated by behavioral tests. Therefore, analgesic drugs affecting this type of pain are more likely to be pharmacologically evaluated based on changes in the rate and quality of these two characteristics (allodynia and hyperalgesia) (Nakamura, Tajima, Kawagoe, Kanai, & Mitsuhata, 2009; Yowtak et al., 2013). In this study, it was found that a once‐injected combination of muscimol and endomorphin‐1, at 15 min after injection, produced a statistically significant reduction in cold allodynia (as demonstrated in Figure 3), but it was not significant compared with administration of each drug individually. However, using a combination of drugs for seven consecutive days led to significant changes in animal pain threshold in comparison with the administration of drugs individually. Behavioral studies showed that a 7‐day intrathecal injection of combinatory drugs could significantly increase the pain threshold in cold and mechanical allodynia at 15 min after injection, and this effect remained till 60 min postinjection. Evaluation of mechanical hyperalgesia confirmed the obtained results.

Some studies confirm that GABA receptors are found in areas of the nervous system that are essential for pain relief. Due to the large dispersion of GABAergic neurons in the brain and spinal cord, the stimulation of these neurons can have different effects, including analgesia or hyperalgesia. It is believed that the stimulation of GABA A receptors in the spinal cord leads to analgesia, whereas GABA A receptors stimulation in areas higher than the spinal cord can cause hyperalgesia (Enna & McCarson, 2006). Coincident with this hypothesis, our study showed that the stimulation of GABA A receptors in the spinal cord decreased the pain threshold.

At the time of injury occurrence, the body is able to release substances such as endogenous endorphins that have an analgesic effect and suppress the pain message before reaching higher levels in the nervous system. Endomorphins are secreted from peptidergic neurons. Their receptors are dose‐dependent (the decrease and increase in receptors depends on the dose used) and, unlike external morphine, endomorphins can be easily decomposed in the body. Endomorphins‐1 and 2 can be extracted and isolated from the human brain and the cow (Przewłocki et al., 1999). Some researchers have also shown that endomorphins concentration and the analgesic effect of them decrease after SCI (Chen & Pan, 2006; Wolfe et al., 2007).

In this study, SCI did not change the NMDA receptor's NR1 subunit expression compared with the control group, and it was also observed that the combination therapy significantly increased the expression level of this subunit in comparison with the control and SCI group. Moreover, seven‐day administration of endomorphin‐1 increased the expression of this subunit. Meanwhile, the injection of muscimol significantly reduced the expression of NR1 subunit compared to the SCI group. In a study, 24 hr after SCI, subtypes NR2A and NR2B had a significant decrease compared to the time before surgery, while there was no meaningful change in the NR1 subunit expression 24 hr postlesion (Brown, Wrathall, Yasuda, & Wolfe, 2004). These results are similar to the results of our experiments. Other studies found that nerve damage increased the amount of NR2B subunit expression, with no impact on the expression of the NR2A subunit and relative decline of NR1 subunit expression (Wilson et al., 2005). The severe sensitivity of NMDA receptors following neuronal damage is acceptable to the majority of researchers. In a study that NMDA receptor's NR1 subunit was removed in the spinal cord neurons, it was observed that in rats lacking NR1 gene, not only NMDA receptor activity was blocked but also symptoms such as severe mechanical and thermal sensitivities erupted. Neurons lacking NR1 subunit showed features such as increase of irritability and activity of excitatory synapses due to increased frequency of action potentials in the spinal cord (Pagadala et al., 2013).

All of these findings point to the fact that following neuronal damage, and all subsequent cycles, from progression to recovery, NMDA receptors, are highly dynamic (Caudle, Perez, Del Valle‐Pinero, & Iadarola, 2005). Although many studies have already been done on the role and importance of the NR2 subunit in the activity of NMDA receptors, the role of the NR1 subunit is still unclear. Henderson, Pittman, & Teskey (2012) concluded that following the induction of high‐frequency stimuli into the neocortex region, enhancement in the GABA A receptor's α2 subunit expression and the NR1 subunit of the NMDA receptor would occur. It was also stated that this increase would occur in the expression of the NR1 subunit in each receptor, and in other words, the number of these subunits per receptor would increase without any change in the number of NMDA receptors. Reduction of antioxidants level or inhibition of antioxidant enzymes can lead to oxidative stress, which may damage cells or even cause cell apoptosis (Vertuani, Angusti, & Manfredini, 2004). It has been proven that oxidative stress contributes to the development of diseases such as neuropathic pain, Alzheimer's and Parkinson's disease (Xu et al., 2008). Since the reactive oxygen species (ROS) activity in the spinal cord and changes in the antioxidant system are important causes of pain and hyperalgesia (Wilson et al., 2005), in this study, the effects of microinjection of muscimol and endomorphin‐1 on the antioxidant system were investigated. Briefly, increased ROS in spinal cord may induce pain by reducing GABA inhibitory influence on spinal dorsal horn neurons that are involved in pain transmission. Therefore, using antioxidants can alter the balance to improve symptoms of pain or using a GABA agonist can fix the defect caused by ROS.

Malondialdehyde is a toxic molecule and is considered as an indicator of oxidative stress that interferes with DNA and proteins and results in leukemia and cellular degradation. MDA level is used as an indicator of lipid peroxidation, which indicates the amount of damage caused by free oxygen radicals (Kerimoğlu et al., 2007). In the present study, MDA level was significantly increased in the SCI and Vehicle groups compared to the control group. Meanwhile, the level of this substance in the group receiving muscimol and endomorphin‐1 was significantly lower than the SCI group. On the other hand, there was no significant difference between the group receiving the combination of drugs and control group. This finding revealed the protective effect of combination therapy on MDA production. Our results indicated a significant decrease in SOD activity of SCI and Vehicle group compared with the control group. As noted, combination therapy could efficiently improve SOD activity nearly to control and sham group. However, there was no significant difference between the group receiving the medication individually (muscimol and endomorphin) with the group receiving the combination of drugs. This suggests the effectiveness of prescribing a combination of drugs to bring the SOD level back to the normal range.

Our results showed that GSH activity was significantly decreased in the SCI group as compared with the control group. In SCI group that received combination therapy, GSH activity efficiently improved nearly to control group and was significantly higher than the SCI group.

Some studies have shown that hippocampal neurons with overexpression of SOD exhibit a higher level of inhibitory and spontaneous synaptic currents. These inhibitory currents inhibit membrane depolarization. The physiological mechanism of this inhibition is due to the upregulation of GABA‐dependent neurotransmission. It has been seen that SOD can directly affect GABA‐dependent neurotransmitter transmissions (Levkovitz, Avignone, Groner, & Segal, 1999).

Treatment with muscimol and endomorphin‐1 in combination and individually markedly increased GSH and SOD activity when compared with the SCI group.

On the other hand, in all figures of oxidative stress, there was no significant difference between the group receiving the drug combination and the normal group. This demonstrates the efficacy of combination drug administration to return MDA, SOD, and GSH levels to normal levels. Therefore, it can be concluded that combination of the drugs is maybe more effective than individual injection of them to cure all the aspects of spinal cord injury such as oxidative stress.

## 5 CONCLUSION

Overall, the results of this study indicate that combination therapy with muscimol and endomorphin‐1 for seven consecutive days can reduce the pain threshold of animals with SCI compared to the administration of each medication individually. Isobologram studies indicated that the interaction of muscimol and endomorphin‐1 is additive. It was also found that simultaneous use of muscimol and endomorphin could improve the reduction in the expression of 2α subunit expression of GABA A receptors after spinal cord injury to normal. Simultaneous use of muscimol and endomorphin‐1 also increases the amount of NR1 subunit from NMDA receptors.

## CONFLICT OF INTEREST

The authors declare that they have no competing interests.

## AUTHOR CONTRIBUTION

Farinaz Nasirinezhad designed the study and involved in fund collection. Marjan Hosseini and Zohreh Karami collected the data. Mahmood Yousefifard contributed to statistical analysis. Marjan Hosseini involved in interpretation of data. Marjan Hosseini and Elham Zamani involved in preparation of the manuscript. Marjan Hosseini and Atousa Janzadenh performed literature search.

## Data Availability

The data that support the findings of this study are available from the corresponding author upon reasonable request.
